# Assessing PD-L1 Expression Status Using Radiomic Features from Contrast-Enhanced Breast MRI in Breast Cancer Patients: Initial Results

**DOI:** 10.3390/cancers13246273

**Published:** 2021-12-14

**Authors:** Roberto Lo Gullo, Hannah Wen, Jeffrey S. Reiner, Raza Hoda, Varadan Sevilimedu, Danny F. Martinez, Sunitha B. Thakur, Maxine S. Jochelson, Peter Gibbs, Katja Pinker

**Affiliations:** 1 Breast Imaging Service, Department of Radiology, Memorial Sloan Kettering Cancer Center, New York, NY 10065, USA; logullor@mskcc.org (R.L.G.); reinerj@mskcc.org (J.S.R.); martind4@mskcc.org (D.F.M.); thakurs@mskcc.org (S.B.T.); jochelsm@mskcc.org (M.S.J.); gibbsp@mskcc.org (P.G.); 2 Department of Pathology, Memorial Sloan Kettering Cancer Center, New York, NY 10065, USA; weny@mskcc.org (H.W.); hodar@ccf.org (R.H.); 3 Department of Epidemiology and Biostatistics, Memorial Sloan Kettering Cancer Center, New York, NY 10017, USA; SevilimS@mskcc.org; 4 Department of Medical Physics, Memorial Sloan Kettering Cancer Center, New York, NY 10065, USA

**Keywords:** radiomics, PD-L1, breast cancer, magnetic resonance imaging

## Abstract

**Simple Summary:**

To our knowledge, this is the first study assessing radiomics coupled with machine learning from MRI-derived features to predict PD-L1 expression status in biopsy-proven triple negative breast cancers and comparing the performance of this approach with the performance of qualitative assessment by two radiologists. This pilot study shows that radiomics analysis coupled with machine learning of DCE-MRI is a promising approach to derive prognostic and predictive information and to select patients who could benefit from anti-PD-1/PD-L1 treatment. This technique could also be used to monitor PD-L1 expression, as it can vary over time and between different regions of the tumor, thus avoiding repeated biopsies.

**Abstract:**

The purpose of this retrospective study was to assess whether radiomics analysis coupled with machine learning (ML) based on standard-of-care dynamic contrast-enhanced magnetic resonance imaging (DCE-MRI) can predict PD-L1 expression status in patients with triple negative breast cancer, and to compare the performance of this approach with radiologist review. Patients with biopsy-proven triple negative breast cancer who underwent pre-treatment breast MRI and whose PD-L1 status was available were included. Following 3D tumor segmentation and extraction of radiomic features, radiomic features with significant differences between PD-L1+ and PD-L1− patients were determined, and a final predictive model to predict PD-L1 status was developed using a coarse decision tree and five-fold cross-validation. Separately, all lesions were qualitatively assessed by two radiologists independently according to the BI-RADS lexicon. Of 62 women (mean age 47, range 31–81), 27 had PD-L1− tumors and 35 had PD-L1+ tumors. The final radiomics model to predict PD-L1 status utilized three MRI parameters, i.e., variance (FO), run length variance (RLM), and large zone low grey level emphasis (LZLGLE), for a sensitivity of 90.7%, specificity of 85.1%, and diagnostic accuracy of 88.2%. There were no significant associations between qualitative assessed DCE-MRI imaging features and PD-L1 status. Thus, radiomics analysis coupled with ML based on standard-of-care DCE-MRI is a promising approach to derive prognostic and predictive information and to select patients who could benefit from anti-PD-1/PD-L1 treatment.

## 1. Introduction

In the last decade, immunotherapy has emerged as a key player in the field of oncology, with encouraging results seen in cancers such as melanoma, renal cell carcinoma, non-small-cell lung cancer, and bladder cancer [1,2,3]. The identification of immunity-related characteristics has also been extended to breast cancer, wherein the presence of tumor-infiltrating lymphocytes (TILs) in breast tumors has been associated with increased survival and complete response rates after neoadjuvant chemotherapy [4,5,6,7].

The programmed cell death 1 receptor/programmed cell death 1 ligand 1 (PD-1/PD-L1) pathway is responsible for maintaining peripheral tolerance and regulating inflammation. An active antitumoral immune response present in the tumor microenvironment can be regulated via negative feedback by the overexpression of PD-L1. PD-L1 is a transmembrane protein that interacts with PD-1 expressed on activated T cells, causing the inhibition of T cell proliferation, cytokine production, and cytolytic activity, consequently leading to the functional inactivation of T cells [8,9,10].

While tumoral overexpression of PD-L1 has a negative influence on survival and treatment response by decreasing the anti-tumoral inflammatory response, the expression of PD-L1 on immune cells and the re-activation of the host’s antitumor immune response through immunotherapy targeting PD-L1 can improve patient outcomes. Breast cancer subtypes, such as triple negative and HER2-positive breast cancers, can overexpress PD-L1 on either breast cancer cells or on TILs. In patients with triple negative breast cancer in particular, the expression of PD-L1 mainly occurs on tumor-infiltrating immune cells rather than on tumor cells [11,12]. Recent trials assessing immunotherapy via PD-L1 blockade in patients with triple negative breast cancer, with the aim of re-activating the host’s antitumor immune response, have shown promising results [13,14,15]. Several other studies have reported that PD-L1 polymorphisms not only significantly influence the breast cancer stage, but also the effectiveness of chemotherapy and overall and disease-free survival after tumor resection [11,14,16].

PD-L1 status is currently assessed using Food and Drug Administration (FDA)-approved companion diagnostic immunohistochemical (IHC) assays. While IHC is the gold standard, it has several limitations. First, inter-test heterogeneity in PDL-1 IHC assessment has been reported due to the differences in the type of antibody used, scoring algorithms, and cut-off points [16]. Second, the expression of PD-1 and PD-L1 can vary over time and between different regions of interest [17]. Third, IHC staining results are usually derived from samples from biopsy or surgical specimens, which may only be a snapshot of a potentially heterogenous tumor [18,19]. Therefore, the development of imaging biomarkers that can enable non-invasive and repeatable assessment of PD-L1 expression derived from the entire tumor is desirable.

High-throughput computational approaches such as advanced machine learning (ML) that can extract numerous characteristics from images of the entire tumor, whether or not they are detectable by the human eye, and that correlate these characteristics with molecular profiles, have heralded a new field of research termed “radiomics”. Radiomic biomarkers have not only demonstrated strong prognostic performance in large cohorts of patients across several cancer types but have also been associated with underlying mutation and gene-expression patterns [20,21]. Radiomic biomarkers can be used for multiple purposes, such as treatment planning [22,23,24,25,26], risk assessment [27,28], and outcome prediction [29,30,31,32,33].

Initial results involving radiomics based on positron emission tomography/computed tomography (PET/CT) or CT imaging to predict PD-L1 expression status in non-small cell lung cancer, oesophageal cancer, and pancreatic cancer have shown promising results [34,35,36]. More recently, there has been a rapid rise in interest in the assessment of PD-L1 expression in breast cancer, especially in aggressive subtypes such as triple negative breast cancer [37,38], where PD-L1 is expressed in up to 20% of cases [12] and may thus potentially serve as a target for PD-1/PD-L1 pathway-directed immunotherapy.

Dynamic contrast-enhanced magnetic resonance imaging (DCE-MRI) is the most sensitive and accurate test for breast cancer diagnosis, characterization, and response assessment. Artificial intelligence (AI)-enhanced DCE-MRI has shown potential to further improve molecular breast cancer subtyping, treatment planning, and treatment monitoring [39]. To date, the potential of radiomics analysis coupled with ML based on DCE-MRI for the prediction of PD-L1 expression status in triple negative breast cancer has not been explored. Thus, the aim of this study was to assess whether radiomics analysis coupled with ML based on standard-of-care DCE-MRI can predict PD-L1 expression status in patients with triple negative breast cancer, and to compare the performance of this approach with radiologist review.

## 2. Materials and Methods

### 2.1. Patient Selection

This was an institutional review board-approved and Health Insurance Portability and Accountability Act-compliant retrospective study for which the need for written informed consent was waived. The study sample consisted of patients with biopsy-proven breast cancer with PD-L1 status assessed using the Ventana PD-L1 SP142 assay who underwent state-of-the-art multiparametric MRI with DCE and T2-weighted imaging using a dedicated breast coil, either at our institution or elsewhere. Breast biopsy was either performed with a vacuum-assisted device or in the form of a spring-loaded core needle biopsy using either ultrasound, stereotactic, or MRI guidance. Multiple samples were taken for each biopsy (usually 4–5 for core needle biopsy and 6–9 for vacuum-assisted biopsy).

Of 156 consecutive patients with PD-L1 SP142 assay assessment, 91 patients were excluded because they did not have a preoperative MRI. Of 65 patients with IHC-proven PD-L1 status and preoperative MRI, three patients were further excluded: one for significant motion between pre-contrast and post-contrast data, one whose MRI protocol was incomplete (missing pre-contrast phase), and one whose study had pre- and post-contrast phases acquired at different matrix sizes. Of the 62 patients included in the final analysis, 39 (63%) had MRI studies performed at our institution and 23 (37%) had studies performed elsewhere.

### 2.2. Breast Imaging Acquisition

Breast MRI examinations were performed on either a 1.5 T (32/62 patients, 52%) or a 3 T scanner (30/62 patients, 48%) using an 8-channel or 16-channel dedicated surface breast coil. Patients underwent a state-of-the-art breast multiparametric MRI protocol in line with international guidelines [40,41,42]. The imaging protocol is summarized in Table 1.

### 2.3. Qualitative Imaging Assessment

Two fellowship-trained breast radiologists (R1: RLG, and R2: JR) with six and seven years of experience, respectively, interpreted the MR images independently, blinded to IHC results.

On post-contrast T1-weighted images, lesion depth (anterior, middle, or posterior depth) was recorded for each lesion, as this has been shown to be correlated with malignancy [43]. Qualitative image assessment was performed according to the Breast Imaging-Reporting and Data System (BI-RADS) lexicon; specifically, lesion shape, margin, and internal enhancement characteristics were assessed for mass lesions, and distribution and type of enhancement were assessed for non-mass enhancements [42]. Lesion size was measured on cranio-caudal, antero-posterior, and latero-medial planes. On T2-weighted images, signal intensity (hypo-, iso-, hyperintense) was recorded. Background parenchymal enhancement and fibroglandular tissue of the contralateral breast were also assessed using maximum intensity projection images and non-fat saturated T1-weighted images, respectively. Peritumoral oedema and paraseptal oedema (considered as diffuse trabecular oedema) were assessed on T2-weighted images as present or absent. Skin invasion was assessed on contrast enhanced T1-weighted images. The axilla was assessed for pathologically enlarged lymph nodes with suspicious morphology including the loss of fatty hilum on T1 non-fat saturated imaging.

### 2.4. Histopathologic Assessment of PD-L1 Expression Status

**Figure 1:**
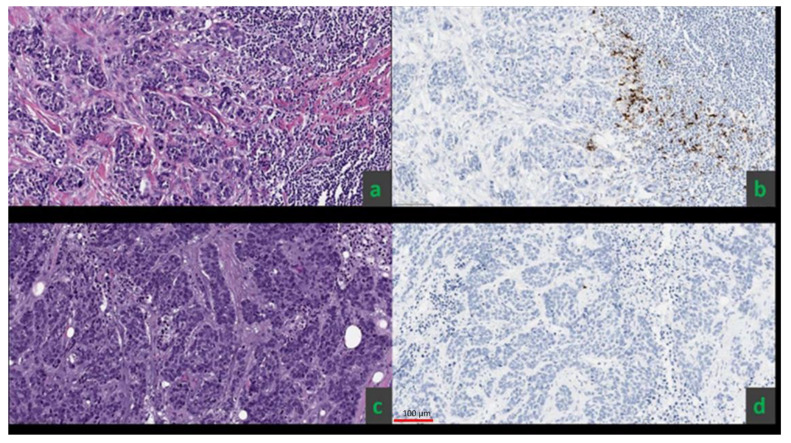
Examples of two invasive ductal carcinomas that were ER, PR, and HER-2 negative (**a**,**c**). The SP142 diagnostic assay yielded positive results for PD-L1 status, defined as ≥1% IC staining (PD-L1 expression on tumor-infiltrating immune cells as a percentage of tumor area) in the first patient (**b**) and negative results in the second patient (**d**).

The VENTANA PD-L1 (SP142) IHC assay was used for the assessment of PD-L1 on formalin-fixed, paraffin-embedded tissue sections from the primary breast cancer or from soft tissue/chest wall recurrence in three patients with a history of ipsilateral mastectomy with reconstruction. PD-L1 positivity was defined as PD-L1 staining in tumor-infiltrating immune cells occupying ≥1% of the tumor area [44] (Figure 1).

### 2.5. Radiomics Analysis

**Figure 2:**
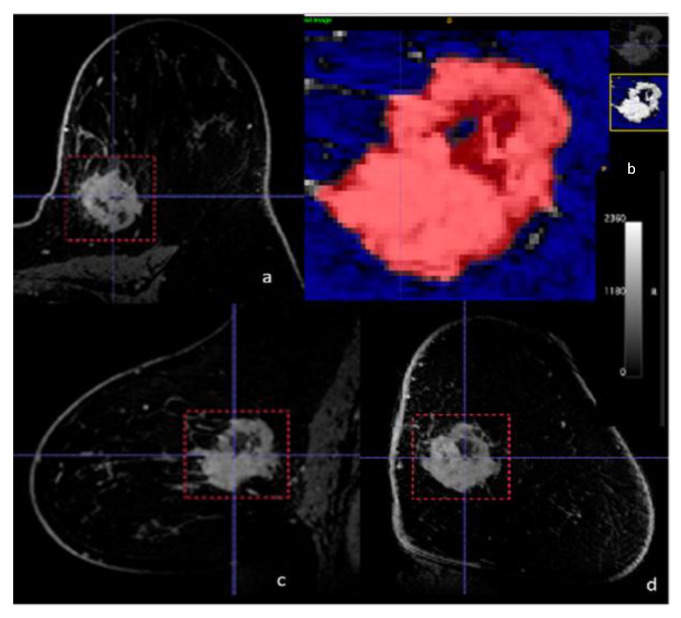
Example of semi-automatic tumor segmentation with dynamic contrast-enhanced MRI—(**a**) axial, (**b**) axial detail, (**c**) sagittal, (**d**) coronal—for radiomics analysis in a 43-year-old female with a biopsy-proven PD-L1-positive poorly differentiated triple negative breast cancer in the 10:00 axis of the left breast.

Digital Imaging and Communications in Medicine (DICOM) images from DCE-MRI and non-contrast T1-weighted imaging were loaded into the open-source image processing tool OsiriX. 3D image segmentation was performed to include the entire enhancing biopsy-proven malignant lesion using the ITK-SNAP software (License: GNU General Public License. 2004) [45]. Semi-automatic tumor segmentation was performed using the soft thresholding mode with the soft binary threshold function, using user-supplied upper and lower threshold values selected by R1 [46]. Since 3D segmentation of the whole tumor was performed, previously biopsied areas were by default included in the whole-tumor ROI. All 3D segmentations were first delineated automatically by means of threshold or clustering, and consequently manually corrected by R1 (Figure 2). Radiomic features extracted from the tumor region of interest (ROI) were calculated automatically.

Relative enhancement maps (% increase in signal from *Pre-* to *Post-Contrast*) were calculated as:

$$100\times \frac{Post\;Contrast-Pre\;Contrast}{Pre\;Contrast}$$

where the post-contrast data were taken as the phase nearest to 2 min post-contrast. Enhancement maps were then reduced to 32 grey levels prior to radiomic feature calculations. Radiomic features were calculated using CERR [47]. One hundred and one features were calculated in six classes (22 first-order, 26 based on grey-level cooccurrence matrices, 16 based on run length matrices, 16 based on size zone matrices, 16 based on neighborhood grey level dependence matrices, and 5 based on neighborhood grey tone difference matrices) (Supplementary Table S1).

To account for potential differences in enhancement characteristics between 1.5 T and 3.0 T scans, ComBat harmonization [48] was performed on the radiomic features to remove site effects (in this study, site effects were due to the two different magnetic field strengths). ComBat harmonization is well-established and has been shown to perform well at removing unwanted inter-site variations [49]. In our study, parametric prior distributions were assumed, and the batch size was 2 (reflecting the two different magnetic field strengths of 1.5 T and 3 T). ComBat uses an empirical Bayes framework to improve the variance of additive and multiplicative site-dependent effects. It estimates a statistical distribution for the parameters of interest by assuming that the data share the same common distribution. Therefore, in our study, information from all cases was used to inform the statistical properties of field strength effects. Harmonization is successful if it removes the noise associated with field strength while maintaining the desired biological variation.

Univariable analysis was then employed to determine features with significant differences between the two groups. The number of features forwarded to model development was then reduced by utilizing correlation analysis. For any pair of parameters that were highly positively correlated (*r* > 0.9) or highly negatively correlated (*r* < −0.9), the parameter with the lowest area under the receiver operating curve (AUROC) was removed. A predictive model was then developed in MATLAB (MathWorks, Natick, MA, USA) using a coarse decision tree and five-fold cross-validation. A coarse decision tree was employed since decision trees are known to be easy to read and interpret. The maximum number of splits employed was limited to four to reduce the possibility of overparameterization. This process was then repeated 1000 times to provide final diagnostic metrics.

### 2.6. Statistical Analysis

Statistical analysis was conducted using SAS (version 9.4, SAS Institute, Cary, NC, USA). Continuous variables were summarized using means (±standard deviation) and medians (range); categorical variables were summarized using proportions. Univariable analysis using the Chi-square test or Fisher’s exact test was performed to assess associations between the qualitatively assessed imaging parameters with PD-L1 expression status (positive vs. negative). The Wilcoxon rank-sum test was used to assess associations between continuous variables and PD-L1 expression status. *p*-values < 0.05 were considered significant. To determine inter-reader agreement, weighted Cohen’s κ was used to assess ordinal parameters, while simple Cohen’s κ was used to assess the inter-reader agreement for nominal parameters.

## 3. Results

### 3.1. Patient Sample and Breast Lesion Characteristics

Of the 62 patients included in the final analysis (mean age 47 ± 11.8; range 31–81), 27 were PD-L1 SP142-negative (PD-L1−) and 35 were PD-L1 SP142-positive (PD-L1+). The average size of lesions measured by R1 in the antero-posterior, latero-medial, and cranio-caudal diameter was 31 × 19 × 31 mm for PD-L1-positive cancers and 27 × 20 × 23 mm for PD-L1-negative cancers (*p* = 0.4–0.9).

### 3.2. Radiomics Analysis and ML

At univariable analysis, only five (out of 101) radiomic features were significantly different between PD-L1− and PD-L1+ patients: variance (from the first order class), run length variance (from the run length matrix class), large zone emphasis (from the size zone matrix class), large zone low grey level emphasis (from the size zone matrix class), and zone level variance (from the size zone matrix class) (Table 2). Table 3 demonstrates strong correlations between large zone emphasis, large zone low grey level emphasis, and zone level variance; thus, utilizing univariable area under the receiver operating curve (AUROC) values, only large zone low grey level emphasis was retained from large zone emphasis, large zone low grey level emphasis, and zone level variance. The final coarse decision tree model therefore included three parameters: variance (from the first order class), run length variance (from the run length matrix class), and large zone low grey level emphasis (from the size zone matrix class). The final model reached a sensitivity of 90.7%, specificity of 85.1%, positive predictive value of 88.8%, negative predictive value of 87.8%, and accuracy in prediction of PD-L1 status of 88.2% (Table 4). By extracting the three stated radiomics features after identical pre-processing and employing the developed decision tree, we were able to assess the validity of the final model.

### 3.3. Quantitative Assessment Using BI-RADS

**Figure 3:**
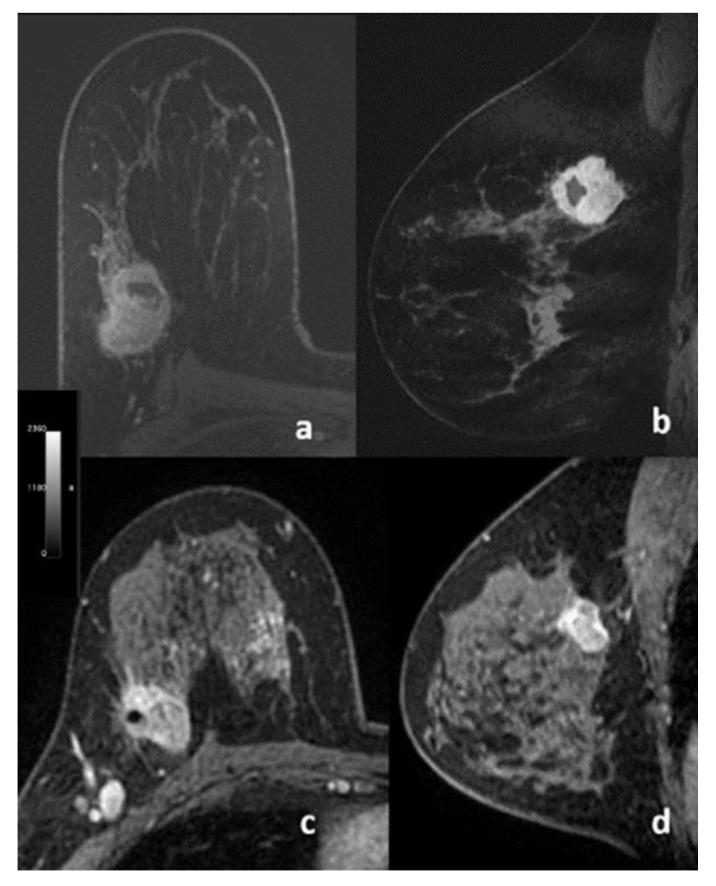
Transverse (**a**,**c**) and sagittal (**b**,**d**) post-contrast dynamic T1-weighted fat-suppressed MR images of two patients with triple negative breast cancer. (**a**,**b**) 33-year-old female with a biopsy-proven, SP142 diagnostic assay PD-L1-positive, poorly differentiated invasive ductal carcinoma. (**c**,**d**) 50-year-old female with a biopsy-proven, SP142 diagnostic assay PD-L1-negative, poorly differentiated invasive ductal carcinoma.

No significant associations were found between any qualitatively assessed lesion parameters and PD-L1 expression status. Table 5 shows the results from univariable analysis according to qualitative assessments by the two radiologists (R1 and R2), with *p*-values ranging from 0.08 to >0.9. Examples are reported in Figure 3. Supplementary Table S2 shows the inter-reader agreement between R1 and R2. For margin assessment, while there was agreement for 44/62 patients, the κ value was only 0.24.

## 4. Discussion

In this feasibility study, we examined whether radiomics analysis coupled with ML based on standard-of-care pre-treatment DCE-MRI can predict PD-L1 status as assessed with PD-L1 SP142 in patients with triple negative breast cancer, and we compared this approach with qualitative radiologist assessment. Results show that DCE-MRI-based radiomics analysis coupled with ML was promising; the final predictive model yielded an accuracy in the prediction of PD-L1 status of 88.2%, sensitivity of 90.7, specificity of 85.1, PPV of 88.8, and NPV of 87.8. On the other hand, no significant associations were found between any of the qualitatively assessed features on DCE-MRI and PD-L1 expression (*p*-values ranging from 0.08 to >0.9).

In the pre-treatment setting, the non-invasive and accurate assessment of PD-L1+ TILs can be valuable to provide predictive information with respect to the amenability of triple negative breast cancer to cytotoxic chemotherapy. Several trials have also demonstrated promising results in breast cancer treatment with immunotherapy targeting PD-L1 blockade [13,14,15]. In addition, PD-L1 status was promising to predict prognosis, as PD-L1+ triple negative breast cancers were associated with significantly better progression-free survival compared with PD-L1− cancers (progression-free survival of 7.2 months vs. 5.5 months).

Currently, PD-L1 status is assessed using FDA-approved companion diagnostic IHC assays. However, it remains desirable to develop imaging biomarkers that can be used to assess PD-L1 expression of the whole tumor non-invasively and at repeated timepoints. Radiomics analysis is non-invasive, relatively fast and easy to perform, and potentially more easily standardized than IHC assays [21]. A robust, validated radiomic-based signature may be used in the future to predict PD-L1 status rapidly after a routine clinical imaging examination. Radiomics assessment can also be repeatedly performed throughout the duration of treatment, without requiring additional invasive testing procedures to obtain tumor tissue from surgery or biopsy, thereby reflecting dynamic PD-L1 expression for treatment stratification and for monitoring the therapeutic efficacy of checkpoint inhibitors to potentially instruct drug replacement and discontinuation. For this reason, we aimed to assess the PD-L1 expression rate through a radiomics-based model in this study.

Recently, a radiomics-based biomarker (Rad score) was shown to predict the presence of tumor-infiltrating CD8 +TILs in hepatocellular carcinoma and to identify potential candidates for immunotherapy [50]. A study by Wen et al. [34] showed moderate performance for PD-L1 prediction in 220 patients with oesophageal cancer using PET/CT data, with an AUC of 0.692 for the validation set. A study by Jiang at al. [35] in a larger cohort of 399 patients with stage I–IV non-small cell lung cancer assessed ML models from CT-, PET-, and combined PET/CT-derived features to predict PD-L1 assessment, showing that CT-derived features achieved the highest predicting efficacy in discriminating PD-L1 expression level higher than either 1% or 50%. As for predictive models based on PET-derived features, the prediction of outcomes was better than a random guess but significantly worse than CT-derived predicting models. When combined with CT-derived features, PET-derived features showed little improvement in distinguishing PD-L1 expression level over 1%, resulting in AUCs of 0.86, 0.62, and 0.85, respectively, for CT, PET, and PET/CT-derived features. With regards to the prediction of strong PD-L1 expression levels (higher than 50%), the AUC score was improved to 0.91, 0.75, and 0.88 for features derived from CT, PET, and PET/CT, respectively. A study by Iwatate et al. [36] in 107 patients with pancreatic cancer showed that PD-L1 predictive models built with imaging features extracted from CT scans demonstrated an AUC of 0.683, sensitivity of 0.417, and specificity of 0.93. Lastly, a study by Yoon et al. [51], whose aim was to assess PD-L1 expression in 153 patients with advanced stage lung adenocarcinoma, found that quantitative CT radiomic features helped predict PD-L1 expression, whereas none of the qualitative imaging findings were associated with PD-L1 expression. The AUC of the Rad-score used to predict PD-L1 expression was 0.667. The results from our study and from others highlight the necessity for the implementation of AI-enhanced imaging to derive better clinical value and to shape the way we care for our patients towards the goal of achieving precision medicine in breast cancer.

Our results show that radiomics analysis and ML can quantify tumor phenotypical differences and provide non-invasive predictive information with regards to PD-L1 expression. When compared with qualitative assessment performed by two experienced radiologists, radiomics coupled with ML was superior to predict PD-L1 positive or negative status. Inter-reader agreement between the two radiologists was low, probably due to the low number of lesions with circumscribed or spiculated margins (seven for R1 and eight for R2) as compared with lesions with irregular margins that were predominant. Our results also show overall better performance of our ML model in the prediction of PD-L1 status compared with previous studies, probably due to the fact that radiomics features were extracted from MRI images rather than PET/CT or CT images in our study. Breast MRI is the most sensitive modality for breast cancer detection [52] and provides not only excellent morphologic information due to higher tissue contrast but also functional information related to vascularization with dynamic imaging; moreover, it is probably the imaging modality for which data for AI studies on breast cancer is most commonly available [39].

This study has several limitations. First, its retrospective nature and relatively small patient cohort precluded a separate validation cohort. However, this is the first imaging study to address this question of the feasibility of radiomics based on pre-treatment standard-of-care DCE-MRI to predict PD-L1 status in patients with triple negative breast cancer. Second, PD-L1 status was assessed using only one PD-L1 IHC assay, namely PD-L1 SP142. Therefore, the results from this study, albeit promising, must be validated in larger prospective studies with different available PD-L1 IHC assays. Another limitation is that ROIs for the radiomics analysis were obtained once. However, it should be noted that ROIs were acquired with semi-automatic segmentation using the soft thresholding mode in ITK-SNAP (license: GNU General Public License. 2004) [45] and consequently they were only manually corrected by R1 if necessary, which rendered this analysis less operator-dependent.

## 5. Conclusions

In conclusion, this feasibility study demonstrated that radiomics analysis coupled with ML based on standard-or-care DCC-MRI can predict PD-L1 status, and thus may be used as a possible alternative to IHC assays to identify patients who could benefit from anti-PD-1/PD-L1 treatment. Meanwhile, the use of standard, qualitatively assessed BI-RADS descriptive characteristics was not predictive of PD-L1 status.

## Figures and Tables

**Table 1:** Summary of imaging sequences and acquisition parameters used in the study.

MR Sequences	Acquisition Parameters *
Axial fat-suppressed 2D T2-weighted imaging	TR, 3500–5500 ms; TE, 100 ms; refocusing flip angle, ‘auto’; slice thickness, 3 mm; gap, 0 mm; field of view, 34–38 cm; matrix size, 320 × 320; bandwidth, 125 kHz for 1.5 T and 83 kHz for 3.0 T; parallel imaging, ‘ASSET’; acceleration factor, 2; acquisition time, 4–5 min
Axial DWI using 2D single-shot with echo-planar imaging (EPI) **	2 b-values (b = 0, 800); TR, 6000 ms; TE, ‘minimum’; flip angle, 90°; field of view, 32–38 cm, matrix size, 128 × 128 (for 1.5 T), 256 × 256 (for 3.0 T); fat suppression, ‘special’; dual shims, ’on’; slice thickness, 4–5 mm; gap, 0 mm; Number of slices, 25–30; parallel imaging, ‘ASSET’; acquisition time, 3 min
Axial non-fat-suppressed 3D T1-weighted imaging	TR, 4–5.2 ms; TE, 2.1–2.4 ms; flip angle, 10°; bandwidth, 100 kHz (1.5 T) and 62.5 kHz (3.0 T); field of view, 32–38 cm; matrix size, 256 × 256 (for 1.5 T) and 320 × 320 (for 3.0 T); slice thickness, 0.8–1.1 mm; gap, 0 mm; fat suppression, no; number of slices, 200–310; parallel imaging, ‘ASSET’; acquisition time, 1.5–2.0 min
Axial fat-suppressed 3D T1-weighted imaging using a Volume Image Breast Assessment (VIBRANT) gradient echo. One sequence before and 3 sequences after intravenous administration of a gadolinium-based contrast agent	TR, 4–5.2 ms; TE, 2.1–2.4 ms; flip angle, 10°; bandwidth, 62.5 kHz; field of view, 32–38 cm; matrix size, 256 × 256 (for 1.5 T) and 320 × 320 (for 3.0 T); slice thickness, 0.8–1.1 mm; gap, 0 mm; fat suppression, yes; number of slices, 200–310; parallel imaging, ‘ASSET’; acquisition time, 1.5–2.0 min per timepoint

* ASSET, array spatial sensitivity encoding technique; TR, repetition time; TE, echo time. ** DWI and ADC maps were available only in 65 patients.

**Table 2:** Mean values of radiomics-derived metrics for tumors and fibroglandular tissue.

Parameter (Class)	PD-L1 Negative	PD-L1 Positive	*p*-Value
Variance (FO)	2370 (−1020 to 210,730)	4030 (840 to 112,620)	0.010
Run length variance (RLM)	0.17 (−0.09 to 4.47)	0.27 (0.11 to 1.11)	0.048
large zone emphasis (SZM)	7.0 (−13.2 to 1144.8)	31.8 (−4.3 to 146.4)	0.003
lzlgle (SZM)	−1.0 (−2.9 to 281.3)	8.0 (−2.0 to 28.9)	<0.001
Zone level variance (SZM)	4.8 (−12.8 to 1107.4)	30.4 (−6.6 to 127.4)	0.003

Abbreviations: FO, first order; RLM, run length matrix; SZM, size zone matrix; lzlgle, large zone low grey level emphasis. Values quoted as median (range).

**Table 3:** Correlation analysis between significant radiomics features, to reduce the number of parameters inputted to the predictive model.

	Spearman Rank Correlation Coefficients					AUROC
	variance	rlv	lze	lzlgle	zlv	
variance		0.537	0.511	0.602	0.506	0.692
rlv	0.537		0.782	0.665	0.770	0.648
lze	0.511	0.782		0.912	0.997	0.723
lzlgle	0.602	0.665	0.912		0.920	0.767
zlv	0.506	0.770	0.997	0.920		0.723

Abbreviations: rlv, run length variance; lze, large zone emphasis; lzlgle, large zone low grey level emphasis; zlv, zone level variance; AUROC, area under the receiver operating curve.

**Table 4:** Diagnostics metrics for the final predictive coarse decision tree model utilizing three radiomics features: variance (from the first order class), run length variance (from the run length matrix class), and large zone low grey level emphasis (from the size zone matrix class).

AUROC	Sensitivity	Specificity	PPV	NPV	Accuracy
0.904	90.7	85.1	88.8	87.8	88.2
(0.82–0.99)	(76.9–98.2)	(66.3–95.8)	(76.3–95.2)	(72.0–95.8)	(78.1–95.3)

Abbreviations: AUROC, area under the receiver operating curve; PPV, positive predictive value; NPV, negative predictive value.

**Table 5:** Univariable analysis according to independent radiologist assessment. * One patient was not assessed because of contralateral mastectomy.

		Reader 1				Reader 2		
Imaging Feature	Overall	PD-L1−	PD-L1+	*p*-Value	Overall	PD-L1−	PD-L1+	*p*-Value
BPE *				0.8				0.7
Minimal	15 (25)	8 (31)	7 (20)		25 (41)	12 (46)	13 (37)	
Mild	26 (43)	11 (42)	15 (43)		16 (26)	5 (19)	11 (31)	
Moderate	14 (23)	5 (19)	9 (26)		7 (11)	3 (12)	4 (11)	
Marked	6 (9.8)	2 (7.7)	4 (11)		13 (21)	6 (23)	7 (20)	
FGT *				0.3				0.6
Almost entirely fat	3 (4.9)	1 (3.8)	2 (5.7)		3 (4.9)	1 (3.8)	2 (5.7)	
Scattered FGT	16 (26)	4 (15)	12 (34)		12 (20)	3 (12)	9 (26)	
Heterogeneous FGT	36 (59)	19 (73)	17 (49)		31 (51)	15 (58)	16 (46)	
Extreme FGT	6 (9.8)	2 (7.7)	4 (11)		15 (25)	7 (27)	8 (23)	
**Depth**				0.11				0.2
Anterior	3 (4.8)	0 (0)	3 (8.6)		5 (8.1)	0 (0)	5 (14)	
Middle	15 (24)	10 (37)	5 (14)		15 (24)	8 (30)	7 (20)	
Posterior	40 (65)	16 (59)	24 (69)		33 (53)	14 (52)	19 (54)	
All depth	4 (6.5)	1 (3.7)	3 (8.6)		9 (15)	5 (19)	4 (11)	
**T2 signal intensity**				0.5				>0.9
Hypointense	4 (6.5)	3 (11)	1 (2.9)		16 (26)	7 (26)	9 (26)	
Isointense	27 (44)	11 (41)	16 (46)		23 (37)	10 (37)	13 (37)	
Hyperintense	31 (50)	13 (48)	18 (51)		23 (37)	10 (37)	13 (37)	
**Peritumoral oedema**				0.9				>0.9
Present	27 (44)	11 (41)	16 (46)		35 (56)	15 (56)	20 (57)	
**Paraseptal oedema**				>0.9				0.6
Present	24 (39)	10 (37)	14 (40)		24 (39)	9 (33)	15 (43)	
**Skin invasion**				>0.9				0.7
Present	4 (6.5)	2 (7.4)	2 (5.7)		7 (11)	4 (15)	3 (8.6)	
**Axillary lymphadenopathy**				0.5				>0.9
Present	34 (55)	13 (48)	21 (60)		38 (61)	16(59)	22 (63)	
**Focality**				0.2				0.4
Unifocal	26 (42)	8 (30)	18 (51)		28 (45)	10 (37)	18 (51)	
Multifocal	10 (16)	6 (22)	4 (11)		8 (13)	3 (11)	5 (14)	
Multicentric	26 (42)	13 (48)	13 (37)		26 (42)	14 (52)	12 (34)	
**BI-RADS**				>0.9				>0.9
4	21 (34)	8 (30)	13 (37)		8 (13)	3 (11)	5 (14)	
5	41 (66)	19 (70)	22 (63)		54 (87)	24 (89)	30 (86)	
**Enhancement type**				0.4				0.3
Mass like	29 (47)	10 (37)	19 (54)		29 (47)	10 (37)	19 (54)	
Non mass like	6 (9.7)	3 (11)	3 (8.6)		8 (13)	5 (19)	3 (8.6)	
Mixed	27 (44)	14 (52)	13 (37)		25 (40)	12 (44)	13 (37)	
**Shape (mass)**				>0.9				>0.9
Oval	1 (1.8)	0 (0)	1 (3.1)		1 (1.9)	0 (0)	1 (3.1)	
Round	0 (0)	0 (0)	0 (0)		3 (5.6)	1 (4.5)	2 (6.2)	
Irregular	55 (98)	24 (100)	31 (97)		50 (93)	21 (95)	29 (91)	
**Margins (mass)**				0.4				0.7
Circumscribed	0 (0)	0 (0)	0 (0)		1 (1.9)	0 (0)	1 (3.1)	
Irregular	49 (88)	20 (83)	29 (91)		46 (85)	18 (82)	28 (88)	
Spiculated	7 (12)	4 (17)	3 (9.4)		7 (13)	4 (18)	3 (9.4)	
**Enhancement (mass)**				0.3				0.4
Homogeneous	0 (0)	0 (0)	0 (0)		0 (0)	0 (0)	0 (0)	
Heterogeneous	47 (84)	20 (83)	27 (84)		37 (69)	17 (77)	20 (62)	
Rim enhancement	9 (16)	4 (17)	5 (16)		17 (31)	5 (23)	12 (38)	
Dark internal septations	0 (0)	0 (0)	0 (0)		0 (0)	0 (0)	0 (0)	
**Distribution (non-mass)**				0.5				0.08
Focal	1 (3)	1 (5.9)	0 (0)		1 (3)	1 (5.9)	0 (0)	
Linear	14 (42)	8 (47)	6 (38)		11 (33)	7 (41)	4 (25)	
Regional	0 (0)	0 (0)	0 (0)		0 (0)	0 (0)	0 (0)	
Segmental	8 (24)	5 (29)	3 (19)		9 (27)	6 (35)	3 (19)	
Multiple regions	9 (27)	3 (18)	6 (38)		5 (15)	0 (0)	5 (31)	
Diffuse	1 (3)	0 (0)	1 (6.2)		7 (21)	3 (18)	4 (25)	
**Enhancement (non-mass)**				0.5				0.4
Homogeneous	3 (9.1)	1 (5.9)	2 (12)		1 (3)	0 (0)	1 (6.2)	
Heterogeneous	11 (33)	7 (41)	4 (25)		20 (61)	12 (71)	8 (50)	
Clumped	18 (55)	8 (47)	10 (62)		12 (36)	5 (29)	7 (44)	
Clustered rings	1 (3)	1 (5.9)	0 (0)		0 (0)	0 (0)	0 (0)	

Abbreviations: BPE, background parenchymal enhancement; FGT, fibroglandular tissue.

## Data Availability

The datasets used and analyzed in this study are not publicly available due to patient privacy requirements but are available upon reasonable request from the corresponding author. The code for radiomic feature extraction used in this study is publicly available via the open-source software CERR (https://github.com/cerr/CERR, last accessed date 12 November 2021).

## Supplementary Materials

The following are available online at https://www.mdpi.com/article/10.3390/cancers13246273/s1, Table S1: Radiomic features; Table S2: Inter-reader agreement.

## References

[1] Alexandrov L.B., Nik-Zainal S., Wedge D.C., Aparicio S.A., Behjati S., Biankin A.V., Bignell G.R., Bolli N., Borg A., Børresen-Dale A.L. (2013). Signatures of mutational processes in human cancer. Nature.

[2] Allison J.P. (2015). Immune Checkpoint Blockade in Cancer Therapy: The 2015 Lasker-DeBakey Clinical Medical Research Award. Jama.

[3] Ohaegbulam K.C., Assal A., Lazar-Molnar E., Yao Y., Zang X. (2015). Human cancer immunotherapy with antibodies to the PD-1 and PD-L1 pathway. Trends Mol. Med..

[4] Loi S., Michiels S., Salgado R., Sirtaine N., Jose V., Fumagalli D., Kellokumpu-Lehtinen P.L., Bono P., Kataja V., Desmedt C. (2014). Tumor infiltrating lymphocytes are prognostic in triple negative breast cancer and predictive for trastuzumab benefit in early breast cancer: Results from the FinHER trial. Ann. Oncol..

[5] Denkert C., Loibl S., Noske A., Roller M., Müller B.M., Komor M., Budczies J., Darb-Esfahani S., Kronenwett R., Hanusch C. (2010). Tumor-associated lymphocytes as an independent predictor of response to neoadjuvant chemotherapy in breast cancer. J. Clin. Oncol..

[6] Ali H.R., Provenzano E., Dawson S.J., Blows F.M., Liu B., Shah M., Earl H.M., Poole C.J., Hiller L., Dunn J.A. (2014). Association between CD8+ T-cell infiltration and breast cancer survival in 12,439 patients. Ann. Oncol..

[7] Pérez-García J.M., Llombart-Cussac A., María G.C., Curigliano G., López-Miranda E., Alonso J.L., Bermejo B., Calvo L., Carañana V., de la Cruz Sánchez S. (2021). Pembrolizumab plus eribulin in hormone-receptor-positive, HER2-negative, locally recurrent or metastatic breast cancer (KELLY): An open-label, multicentre, single-arm, phase II trial. Eur. J. Cancer.

[8] Guleria I., Khosroshahi A., Ansari M.J., Habicht A., Azuma M., Yagita H., Noelle R.J., Coyle A., Mellor A.L., Khoury S.J. (2005). A critical role for the programmed death ligand 1 in fetomaternal tolerance. J. Exp. Med..

[9] Kula A., Dawidowicz M., Kiczmer P., Prawdzic Seńkowska A., Świętochowska E. (2020). The role of genetic polymorphism within PD-L1 gene in cancer. Review. Exp. Mol. Pathol..

[10] Paver E.C., Cooper W.A., Colebatch A.J., Ferguson P.M., Hill S.K., Lum T., Shin J.S., O’Toole S., Anderson L., Scolyer R.A. (2021). Programmed death ligand-1 (PD-L1) as a predictive marker for immunotherapy in solid tumours: A guide to immunohistochemistry implementation and interpretation. Pathology.

[11] Sabatier R., Finetti P., Mamessier E., Adelaide J., Chaffanet M., Ali H.R., Viens P., Caldas C., Birnbaum D., Bertucci F. (2015). Prognostic and predictive value of PDL1 expression in breast cancer. Oncotarget.

[12] Mittendorf E.A., Philips A.V., Meric-Bernstam F., Qiao N., Wu Y., Harrington S., Su X., Wang Y., Gonzalez-Angulo A.M., Akcakanat A. (2014). PD-L1 expression in triple-negative breast cancer. Cancer Immunol. Res..

[13] Adams S., Loi S., Toppmeyer D., Cescon D.W., De Laurentiis M., Nanda R., Winer E.P., Mukai H., Tamura K., Armstrong A. (2019). Pembrolizumab monotherapy for previously untreated, PD-L1-positive, metastatic triple-negative breast cancer: Cohort B of the phase II KEYNOTE-086 study. Ann. Oncol..

[14] Schmid P., Adams S., Rugo H.S., Schneeweiss A., Barrios C.H., Iwata H., Diéras V., Hegg R., Im S.A., Shaw Wright G. (2018). Atezolizumab and Nab-Paclitaxel in Advanced Triple-Negative Breast Cancer. N. Engl. J. Med..

[15] Mavratzas A., Seitz J., Smetanay K., Schneeweiss A., Jäger D., Fremd C. (2020). Atezolizumab for use in PD-L1-positive unresectable, locally advanced or metastatic triple-negative breast cancer. Future Oncol..

[16] Huang W., Ran R., Shao B., Li H. (2019). Prognostic and clinicopathological value of PD-L1 expression in primary breast cancer: A meta-analysis. Breast Cancer Res. Treat..

[17] Barrett M.T., Lenkiewicz E., Malasi S., Basu A., Yearley J.H., Annamalai L., McCullough A.E., Kosiorek H.E., Narang P., Wilson Sayres M.A. (2018). The association of genomic lesions and PD-1/PD-L1 expression in resected triple-negative breast cancers. Breast Cancer Res..

[18] Bai H.X., Lee A.M., Yang L., Zhang P., Davatzikos C., Maris J.M., Diskin S.J. (2016). Imaging genomics in cancer research: Limitations and promises. Br. J. Radiol..

[19] Sobral-Leite M., Van de Vijver K., Michaut M., van der Linden R., Hooijer G.K.J., Horlings H.M., Severson T.M., Mulligan A.M., Weerasooriya N., Sanders J. (2018). Assessment of PD-L1 expression across breast cancer molecular subtypes, in relation to mutation rate, BRCA1-like status, tumor-infiltrating immune cells and survival. Oncoimmunology.

[20] Aerts H.J., Velazquez E.R., Leijenaar R.T., Parmar C., Grossmann P., Carvalho S., Bussink J., Monshouwer R., Haibe-Kains B., Rietveld D. (2014). Decoding tumour phenotype by noninvasive imaging using a quantitative radiomics approach. Nat. Commun..

[21] Lambin P., Leijenaar R.T.H., Deist T.M., Peerlings J., de Jong E.E.C., van Timmeren J., Sanduleanu S., Larue R., Even A.J.G., Jochems A. (2017). Radiomics: The bridge between medical imaging and personalized medicine. Nat. Rev. Clin. Oncol..

[22] Ha R., Chin C., Karcich J., Liu M.Z., Chang P., Mutasa S., Pascual Van Sant E., Wynn R.T., Connolly E., Jambawalikar S. (2019). Prior to Initiation of Chemotherapy, Can We Predict Breast Tumor Response? Deep Learning Convolutional Neural Networks Approach Using a Breast MRI Tumor Dataset. J. Digit. Imaging.

[23] Lo Gullo R., Eskreis-Winkler S., Morris E.A., Pinker K. (2020). Machine learning with multiparametric magnetic resonance imaging of the breast for early prediction of response to neoadjuvant chemotherapy. Breast.

[24] Tahmassebi A., Wengert G.J., Helbich T.H., Bago-Horvath Z., Alaei S., Bartsch R., Dubsky P., Baltzer P., Clauser P., Kapetas P. (2019). Impact of Machine Learning With Multiparametric Magnetic Resonance Imaging of the Breast for Early Prediction of Response to Neoadjuvant Chemotherapy and Survival Outcomes in Breast Cancer Patients. Invest. Radiol..

[25] Liu Z., Li Z., Qu J., Zhang R., Zhou X., Li L., Sun K., Tang Z., Jiang H., Li H. (2019). Radiomics of Multiparametric MRI for Pretreatment Prediction of Pathologic Complete Response to Neoadjuvant Chemotherapy in Breast Cancer: A Multicenter Study. Clin. Cancer Res..

[26] Bitencourt A.G.V., Gibbs P., Rossi Saccarelli C., Daimiel I., Lo Gullo R., Fox M.J., Thakur S., Pinker K., Morris E.A., Morrow M. (2020). MRI-based machine learning radiomics can predict HER2 expression level and pathologic response after neoadjuvant therapy in HER2 overexpressing breast cancer. EBioMedicine.

[27] Dembrower K., Liu Y., Azizpour H., Eklund M., Smith K., Lindholm P., Strand F. (2020). Comparison of a Deep Learning Risk Score and Standard Mammographic Density Score for Breast Cancer Risk Prediction. Radiology.

[28] Yala A., Lehman C., Schuster T., Portnoi T., Barzilay R. (2019). A Deep Learning Mammography-based Model for Improved Breast Cancer Risk Prediction. Radiology.

[29] Guo W., Li H., Zhu Y., Lan L., Yang S., Drukker K., Morris E., Burnside E., Whitman G., Giger M.L. (2015). Prediction of clinical phenotypes in invasive breast carcinomas from the integration of radiomics and genomics data. J. Med. Imaging (Bellingham).

[30] Bismeijer T., van der Velden B.H.M., Canisius S., Lips E.H., Loo C.E., Viergever M.A., Wesseling J., Gilhuijs K.G.A., Wessels L.F.A. (2020). Radiogenomic Analysis of Breast Cancer by Linking MRI Phenotypes with Tumor Gene Expression. Radiology.

[31] Mehta S., Hughes N.P., Li S., Jubb A., Adams R., Lord S., Koumakis L., van Stiphout R., Padhani A., Makris A. (2016). Radiogenomics Monitoring in Breast Cancer Identifies Metabolism and Immune Checkpoints as Early Actionable Mechanisms of Resistance to Anti-angiogenic Treatment. EBioMedicine.

[32] Yamamoto S., Maki D.D., Korn R.L., Kuo M.D. (2012). Radiogenomic analysis of breast cancer using MRI: A preliminary study to define the landscape. AJR Am. J. Roentgenol..

[33] Dietzel M., Baltzer P.A., Dietzel A., Vag T., Gröschel T., Gajda M., Camara O., Kaiser W.A. (2010). Application of artificial neural networks for the prediction of lymph node metastases to the ipsilateral axilla-initial experience in 194 patients using magnetic resonance mammography. Acta Radiol..

[34] Wen Q., Yang Z., Zhu J., Qiu Q., Dai H., Feng A., Xing L. (2020). Pretreatment CT-Based Radiomics Signature as a Potential Imaging Biomarker for Predicting the Expression of PD-L1 and CD8+TILs in ESCC. Onco Targets Ther..

[35] Jiang M., Sun D., Guo Y., Guo Y., Xiao J., Wang L., Yao X. (2020). Assessing PD-L1 Expression Level by Radiomic Features From PET/CT in Nonsmall Cell Lung Cancer Patients: An Initial Result. Acad. Radiol..

[36] Iwatate Y., Hoshino I., Yokota H., Ishige F., Itami M., Mori Y., Chiba S., Arimitsu H., Yanagibashi H., Nagase H. (2020). Radiogenomics for predicting p53 status, PD-L1 expression, and prognosis with machine learning in pancreatic cancer. Br. J. Cancer.

[37] Stovgaard E.S., Dyhl-Polk A., Roslind A., Balslev E., Nielsen D. (2019). PD-L1 expression in breast cancer: Expression in subtypes and prognostic significance: A systematic review. Breast Cancer Res. Treat..

[38] Stovgaard E.S., Nielsen D., Hogdall E., Balslev E. (2018). Triple negative breast cancer-prognostic role of immune-related factors: A systematic review. Acta Oncol..

[39] Meyer-Bäse A., Morra L., Meyer-Bäse U., Pinker K. (2020). Current Status and Future Perspectives of Artificial Intelligence in Magnetic Resonance Breast Imaging. Contrast Media Mol. Imaging.

[40] Mann R.M., Kuhl C.K., Kinkel K., Boetes C. (2008). Breast MRI: Guidelines from the European Society of Breast Imaging. Eur. Radiol..

[41] Mann R.M., Balleyguier C., Baltzer P.A., Bick U., Colin C., Cornford E., Evans A., Fallenberg E., Forrai G., Fuchsjäger M.H. (2015). Breast MRI: EUSOBI recommendations for women’s information. Eur. Radiol..

[42] Morris E.A., Comstock C.E., Lee C.H. (2013). ACR BI-RADS^®^ Magnetic Resonance Imaging. ACR BI-RADS^®^ Atlas, Breast Imaging Reporting and Data System.

[43] Marino M.A., Riedl C.C., Bernathova M., Bernhart C., Baltzer P.A.T., Helbich T.H., Pinker K. (2018). Imaging Phenotypes in Women at High Risk for Breast Cancer on Mammography, Ultrasound, and Magnetic Resonance Imaging Using the Fifth Edition of the Breast Imaging Reporting and Data System. Eur. J. Radiol.

[44] Hoda R.S., Brogi E., Dos Anjos C.H., Grabenstetter A., Ventura K., Patil S., Selenica P., Weigelt B., Reis-Filho J.S., Traina T. (2020). Clinical and pathologic features associated with PD-L1 (SP142) expression in stromal tumor-infiltrating immune cells of triple-negative breast carcinoma. Mod. Pathol..

[45] Besson F.L., Henry T., Meyer C., Chevance V., Roblot V., Blanchet E., Arnould V., Grimon G., Chekroun M., Mabille L. (2018). Rapid Contour-based Segmentation for (18)F-FDG PET Imaging of Lung Tumors by Using ITK-SNAP: Comparison to Expert-based Segmentation. Radiology.

[46] Yushkevich P.A., Piven J., Hazlett H.C., Smith R.G., Ho S., Gee J.C., Gerig G. (2006). User-guided 3D active contour segmentation of anatomical structures: Significantly improved efficiency and reliability. Neuroimage.

[47] Apte A.P., Iyer A., Crispin-Ortuzar M., Pandya R., van Dijk L.V., Spezi E., Thor M., Um H., Veeraraghavan H., Oh J.H. (2018). Technical Note: Extension of CERR for computational radiomics: A comprehensive MATLAB platform for reproducible radiomics research. Med. Phys..

[48] Johnson W.E., Li C., Rabinovic A. (2007). Adjusting batch effects in microarray expression data using empirical Bayes methods. Biostatistics.

[49] Fortin J.P., Parker D., Tunç B., Watanabe T., Elliott M.A., Ruparel K., Roalf D.R., Satterthwaite T.D., Gur R.C., Gur R.E. (2017). Harmonization of multi-site diffusion tensor imaging data. Neuroimage.

[50] Liao H., Zhang Z., Chen J., Liao M., Xu L., Wu Z., Yuan K., Song B., Zeng Y. (2019). Preoperative Radiomic Approach to Evaluate Tumor-Infiltrating CD8(+) T Cells in Hepatocellular Carcinoma Patients Using Contrast-Enhanced Computed Tomography. Ann. Surg. Oncol..

[51] Yoon J., Suh Y.J., Han K., Cho H., Lee H.J., Hur J., Choi B.W. (2020). Utility of CT radiomics for prediction of PD-L1 expression in advanced lung adenocarcinomas. Thorac. Cancer.

[52] Zhang L., Tang M., Min Z., Lu J., Lei X., Zhang X. (2016). Accuracy of combined dynamic contrast-enhanced magnetic resonance imaging and diffusion-weighted imaging for breast cancer detection: A meta-analysis. Acta Radiol..

