# Supplementary material for: Assessing PD-L1 Expression Status Using Radiomic Features from Contrast-Enhanced Breast MRI in Breast Cancer Patients: Initial Results
Source: Cancers (Basel). 2021 Dec 14;13(24):6273. doi: 10.3390/cancers13246273 (PMC8699819; doi:10.3390/cancers13246273)
Supplement: Supplementary file 1 [file cancers-13-06273-s001.zip › cancers-1461385-supplementary-revised.pdf]

SUPPLEMENTARY DATA

Contents

Table S1. Radiomic Parameters 2

Table S2. Inter-reader agreement 6

Table S1. Radiomic Parameters

**<u>First Order Parameters:</u> These describe the grey level intensity distribution within the ROI. No account is taken of the spatial relationships between pixels.**
Minimum intensity
Maximum intensity
Mean intensity
Range of intensity values
Standard deviation of intensity values
Variance of intensity values
Median of intensity values
Skewness
Kurtosis
Entropy
Root mean square
Energy
Total energy
Mean absolute deviation
Median absolute deviation
10th percentile intensity value
90th percentile intensity value
Robust mean absolute deviation
Robust median absolute deviation
Inter quartile range
Coefficient of dispersion
Coefficient of variation
**Total: 22 parameters**
**<u>Grey Level Cooccurrence Matrices Based Parameters:</u> These describe the relationships between pixels that are directly next to each other in the image**
Energy
Joint entropy
Joint maximum
Joint average
Joint variance
Contrast
Inverse difference moment
Inverse difference moment normalized
Inverse difference
Inverse difference normalized
Invariance
Dissimilarity

Difference entropy<br>Difference variance<br>Difference average<br>Sum average<br>Sum variance<br>Sum entropy<br>Correlation<br>Cluster tendency<br>Cluster shade<br>Cluster prominence<br>Haralick correlation<br>Auto correlation<br>Information measure of correlation 1<br>Information measure of correlation 2<br>**Total: 26 parameters**
**<u>Run Length Matrices Based Features:</u> These describe the extent of runs of pixels in a straight line with the same intensity. Various parameters are weighted towards emphasizing the presence of short/long runs, low/high grey levels, and combinations thereof.**<br>Grey level non-uniformity<br>Grey level non-uniformity normalized<br>Grey level variance<br>High grey level run emphasis<br>Low grey level run emphasis<br>Long run emphasis<br>Long run high grey level emphasis<br>Long run low grey level emphasis<br>Run emphasis<br>Run length non-uniformity<br>Run length non-uniformity normalized<br>Run length variance<br>Run percentage<br>Short run emphasis<br>Short run high grey level emphasis<br>Short run low grey level emphasis<br>**Total: 16 parameters**
**<u>Size Zone Matrices Based Parameters:</u> These describe the extent of areas of pixels with the same intensity that are connected. Various parameters are weighted towards emphasizing the presence of small/large zones, low/high grey levels, and combinations thereof.**<br>Small zone emphasis<br>Large zone emphasis

Grey level non-uniformity<br>Grey level non-uniformity normalized<br>Size zone non-uniformity<br>Size zone non-uniformity normalized<br>Zone percentage<br>Low grey large zone emphasis<br>High grey large zone emphasis<br>Small zone low grey level emphasis<br>Small zone high grey level emphasis<br>Large zone low grey level emphasis<br>Large zone high grey level emphasis<br>Grey level variance<br>Size zone variance<br>Zone emphasis<br>**Total: 16 parameters**
**<u>Neighborhood Gray Level Difference Matrices Based Parameters:</u> These describe features related to the difference in intensity between a pixel and all its nearest neighbors**<br>Low dependence emphasis<br>High dependence emphasis<br>Low grey level count emphasis<br>High grey level count emphasis<br>Low dependence low grey emphasis<br>Low dependence high grey emphasis<br>High dependence low grey emphasis<br>High dependence high grey emphasis<br>Grey level non-uniformity<br>Grey level non-uniformity normalized<br>Dependence count non-uniformity<br>Dependence count non-uniformity normalized<br>Dependence count percentage<br>Grey level variance<br>Dependence count entropy<br>Entropy<br>Energy<br>**Total: 17 parameters**
**<u>Neighborhood Grey Tone Differences Matrices Based Parameters:</u> These describe features based on a matrix calculated from the intensity differences between a pixel and its neighbors within a set distance**<br>Coarseness<br>Contrast<br>Busyness

Complexity<br>Texture strength<br>**Total: 5 parameters**

Table S2. Inter-reader agreement

kappa	p-value	Factor
0.683	3.92E-08	Depth
0.575	3.67E-06	Contralateral BPE
0.818	2.01E-11	Contralateral FGT
0.745	4.37E-09	Enhancement type
0.718	4.21E-08	Mass shape
0.242	0.076512996	Mass margins
0.476	0.000157161	Mass internal enhancement
0.710	7.20E-05	Non-mass enhancement distribution
0.621	0.000350431	Non-mass internal enhancement
0.470	2.50E-05	T2 signal
0.556	6.06E-06	Peri tumor edema
0.728	9.88E-09	Para septal edema
0.703	6.78E-09	Skin invasion
0.736	4.98E-09	Axillary lymphadenopathy
0.830	6.07E-11	Focality
0.449	2.29E-05	Birads
